# A Novel Method for Estimating Distances from a Robot to Humans Using Egocentric RGB Camera

**DOI:** 10.3390/s19143142

**Published:** 2019-07-17

**Authors:** Sai Krishna Pathi, Andrey Kiselev, Annica Kristoffersson, Dirk Repsilber, Amy Loutfi

**Affiliations:** 1 Center for Applied Autonomous Sensor Systems (AASS), School of Natural Science and Technology, Örebro University, 701 82 Örebro, Sweden; 2 School of Innovation, Design and Engineering, Mälardalen University, 721 23 Västerås, Sweden; 3 School of Medical Sciences, Örebro University, Campus USÖ, 701 82 Örebro, Sweden

**Keywords:** distance estimation, Human–Robot Interaction, social interaction, single RGB image

## Abstract

Estimating distances between people and robots plays a crucial role in understanding social Human–Robot Interaction (HRI) from an egocentric view. It is a key step if robots should engage in social interactions, and to collaborate with people as part of human–robot teams. For distance estimation between a person and a robot, different sensors can be employed, and the number of challenges to be addressed by the distance estimation methods rise with the simplicity of the technology of a sensor. In the case of estimating distances using individual images from a single camera in a egocentric position, it is often required that individuals in the scene are facing the camera, do not occlude each other, and are fairly visible so specific facial or body features can be identified. In this paper, we propose a novel method for estimating distances between a robot and people using single images from a single egocentric camera. The method is based on previously proven 2D pose estimation, which allows partial occlusions, cluttered background, and relatively low resolution. The method estimates distance with respect to the camera based on the Euclidean distance between ear and torso of people in the image plane. Ear and torso characteristic points has been selected based on their relatively high visibility regardless of a person orientation and a certain degree of uniformity with regard to the age and gender. Experimental validation demonstrates effectiveness of the proposed method.

## 1. Introduction

**Figure 1:**
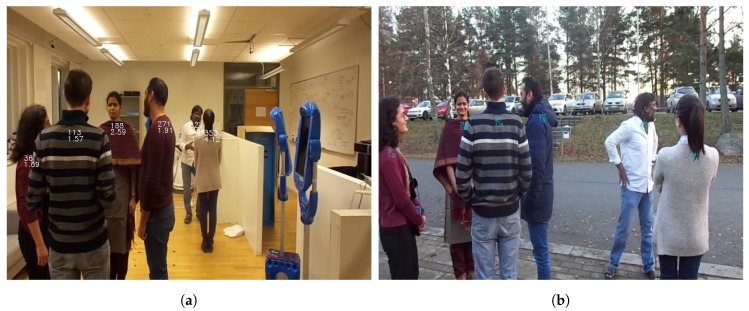
Two sample images of social interactions from an egocentric view with a cluttered background and foreground. Six people are interacting in two groups. Two people, one in each group, are not facing the camera. In (**a**), the estimated location of the torso in the image plane and the observed distance are provided for each person. In (**b**), estimating distance in outdoor environment. The numbers present on the people in the images represent two information’s. The upper number is the location (column number) of the torso of the person in the image. The below number is the distance of the person from the camera in meters.

Spatial placement of actors plays a crucial role in Human–Human Interaction (HHI). Unrestricted by physical constraints or task at hand, it characterizes and influences social relationships between actors. Two widely known theories in social HHI are interpersonal distances (proxemics) [1] and F-formation system [2,3]. These theories show that the spatial relationships between humans depend on their interactions. Humans tend to position themselves at different interpersonal distances and spatial configurations depending on the context. For example, a group of humans interacting with each other can form a circle configuration. Likewise, work in Human–Robot Interaction (HRI) has focused on importing aspects from HHI to create conducive interactions, where robots should adhere to proxemics and F-formations [4]. However, it can be non-trivial for an autonomous or semi-autonomous robot to respect spatial configurations [5]. On the one hand, the robot needs to estimate the distance between itself and persons in the scene using an egocentric perspective in order to determine the opportunities for social interaction, and on the other hand, the robot must be able to join and collaborate with humans. Estimating the distance is difficult for several reasons. First, sensors such as infrared, laser, or ultrasonic, depend on surface uniformity and material to estimate the distance [6,7]. Second, there can be several people in a given scene, and more than one group of people engaged in social interactions at the same time as perceived from the robot’s egocentric perspective (see Figure 1). The fact that people may be occluding each other can negatively affect the accuracy of the distance estimation as well. In many HRI scenarios, robots perceive the scene from an egocentric view often through inbuilt Red–Green–Blue (RGB) cameras [8,9]. Therefore, approaches providing information from single cameras can be relevant to many robotic platforms and Mobile Robotic Telepresence (MRP) robots [5].

For detecting social group interactions, estimating distances to people from an egocentric view in images, videos, or in live feeds is a critical and important information. Different kinds of sensors can be deployed to measure or estimate these distances, for example using a live feed [10,11,12]. For videos, training algorithms could be used [13,14] to estimate distance. However, none of these sensors or training algorithms, work on single images.

In this paper, we present a method to estimate distances from an egocentric camera of a robot to people, considering different complex scenarios that include cluttered background and foreground, when some parts of the body is occluded by other people in the scene, and when a person is not facing the camera.

The main contributions of the work are summarized as follows:

- The formulation of the method using a single RGB image to calculate the distance between the people and a camera source i.e., standalone or on a robot.
- The use of a Euclidean distance measure between characteristic points on the human body as a relationship to the real distance between the people and the camera on the robot.
- An evaluation of results through experiments and tests on the collected datasets that estimate the distance between the people and the camera in the scene.

The remainder of this paper is organized as follows: Section 2 gives an overview of previous works and methods used for estimating distance. Section 3 presents our vision-based method for estimating the distance between people and a camera in detail. Section 4 provides information on how the method was validated. The results of the experimental validation are presented in Section 5. Finally, the applicability of the method within RGB and future works are discussed in Section 6.

## 2. Related Works

The related works to our approach can be categorized into two groups: vision-based methods and non-vision-based methods. Vision-based methods use a camera to capture RGB images and estimate the distance *d* using single or a series of images. Non-vision-based methods use other types of sensors to measure the distance *d* directly, and may include laser range scanners [15,16], sonars [17], infrared sensors [10], ultrasonic sensors [11], etc. Recently, also RGB–Depth (RGB-D) sensors are becoming rapidly popular in robotics for acquiring the depth map in a scene [18,19]. For example, Komatsubara et al. [18] used 24 depth sensors (Kinect) and six cameras to cover an 8 by 16 m area of a room and estimate distances to persons. Depth sensors are used to acquire positional information of people, and the corresponding camera is used to acquire RGB images for face identification. Another example includes Alletto et al. [13] who employs facial landmarks to compute a head pose estimation and build a feature vector to train Random Regression Forests (RRF) using the ground-truth depth data obtained from a RGB-D sensor. While the RGB-D sensors demonstrate good performance in many indoor applications, the performance is generally poor when applied in outdoor environments [19]. Also, the accuracy of this form of distance estimation highly depends on surface uniformity and material [6,7]. Finally, a further shortcoming is that many robots aimed to support social interaction, e.g., most telepresence robots are in fact not equipped with RGB-D sensors.

Vision-based methods use only cameras to capture the RGB images of the scene to compute the distances. There are methods using images for capturing the distance between any object and the camera [20,21]. However, none of them rely on RGB images from single camera as would be the situation for, e.g., some of the social robots and most of the telepresence robots engaged in social interaction with humans. Nedevschi et al. [20] use a stereo vision camera. Suh et al. [21] captures two RGB images from two different positions using a mono camera. There are several methods to estimate the distance without any prior information. Shoani et al. [6] use the size of the face for determining the distance between the person and the camera. This approach works at distances up to 6 m. Konig et al. [22] uses eye to eye distance to estimate the distance between a smart phone camera and a face. This method works for short distances, i.e., less than 90 cm. Konig et al. uses the area of the detected face region to measure the distance between the face and the phone [22]. Work by Burgos-Artizzu et al. [23] uses regression to estimate the distance to a person based on automatically estimated positions of face and head landmarks in images. Recently, deep learning approach has also been used for distance estimation. Bianco et al. [14] estimates distance based on the size of portrayed subjects. The authors train a Convolutional Neural Network (CNN) to predict the size of people portrayed in an image as a proxy for their real-life size. However, the authors provide information on the focal length of the camera externally. The aforementioned methods [6,22,23] provide support in the claim that the distance between people and a camera using single RGB images is related to the distance between certain characteristic features on the human body (in this case the face) and that this distance changes in relationship to the distance from a person to a camera. However, the above referenced methods use frontal face features and in some cases use features related to a person’s facial profile. In social interactions however, people can be facing any direction. Thus, if a robot is to successfully join groups of people, the robot needs to distinguish between different groups of people, regardless in which direction a person may be facing. For example, in a circular formation, the robot’s camera may detect the frontal face of a person, while only seeing the right side, left side or the back of the other people in the formation as shown in Figure 1. The approaches presented in [6,23] would unfortunately fail for 4 people in the scenario, as their frontal face is not at all visible.

The method proposed in this paper estimates distance irrespective of the person’s head pose. The method is based on the perspective distortion principle, e.g., that objects closer to the camera appear larger in the image plane, while objects further from the camera appear smaller. This principle in combination with basic proportions of the human body, lead to a distance estimation.

There are a few areas on the human body which could be captured always regardless of a pose. In our work, we rely on characteristic points that are stable irrespective of whether a person is facing the camera. The selection of these points is performed to (1) adhere to principles of human anatomy, (2) possibility to be detected irrespective of pose. At the same time, the characteristic points should not be too close to each other as this would result in an increased amount of noise in the measurements.

Based on this reasoning, this paper therefore assumes that on an image, the Euclidean distance between an *ear* and a *torso* points is related to the distance from a camera to the person. Additionally, to the aforementioned factors, these characteristic points remain fairly stable with the human growth, in comparison to other body proportions. Furthermore, the accuracy of the method can be improved based on known proportions-change-with-growth models [24]. Based on the assumption that the ear-torso distance is a predictor of the distance to a person, we built up a mathematical form, in which the expected dependency between the ear-torso measurement and the real distance can be described as follows:

(1) $$\begin{array}{cc} D_{et} & =a+\frac{k}{d-b} \end{array}$$

where $D_{et}$ is Euclidean distance between a subject’s ear and torso in the image plane, *d* is the true distance from the camera to the torso point, and *k* is a coefficient accounting for the difference in measurement units (*m* for *d* and *pixels* for $D_{et}$). *a* and *b*, which are correction coefficients along the X- and Y-axis respectively equal to 0.

The method presented in this paper can be used with robots equipped with a single RGB sensor to estimate the distances to humans. At the same time, robots equipped with other types of sensor could benefit from performing a multi-sensor fusion approach to estimate distances more reliably.

## 3. Method

**Figure 2:**
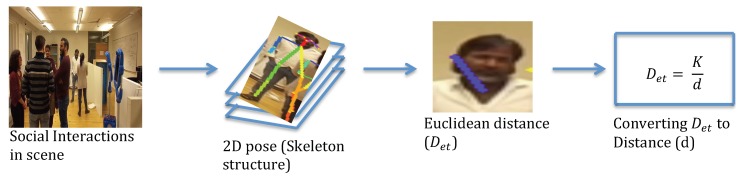
Overview of our method. First, an RGB image is used as input for our method. Second, the 2D pose (skeleton structure) for each person in the scene is obtained using the [25] approach. Third, the Euclidean distance $D_{et}$ between the Ear-torso points is calculated. Finally, real distance *d* is calculated by substituting $D_{et}$ value in Equation (1).

The proposed method is based on using the effect of perspective distortion from the camera’s point of view. Assuming that the Euclidean distance between characteristic points of an object is related to the distance from a camera to the object, the first step in the method is to estimate the position of characteristic points in the image plane. In the second step, the Euclidean distance $D_{et}$ is calculated between these points. Thereafter, the true distance *d* is calculated using Equation (1). Figure 2 provides an overview of the method.

**Figure 3:**
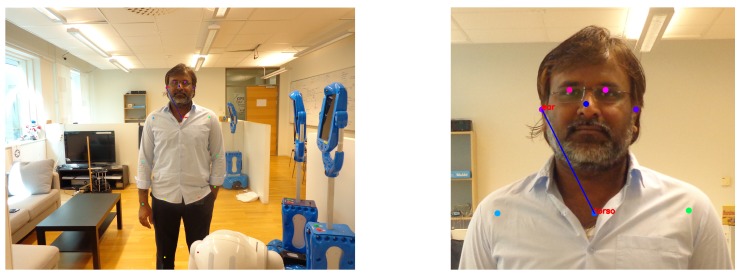
The characteristic points using the method reported in [25]. The left image presents all the characteristic points with colored dots. The right image is the close-up of the corresponding image. The image presents the ear and torso points connected through a line.

There are multiple options for finding characteristic points in images. Those range from using various Computer Vision (CV) techniques (e.g., the Hough transform) to Machine Learning (ML). In this study, we apply the method reported upon in [25] which presents with the characteristic points as shown in Figure 3. This method allows for fitting skeleton models to people in a scene and provides the positions of important characteristic points of a human body. The algorithm learns to relate the limbs or body parts to the respective person in the image using the Part Affinity Fields (PAFs) feature representation. The algorithm jointly learns parts detection and parts association. Thereafter, a greedy parsing algorithm is introduced to produce high quality parses of body poses which achieves real-time performance, irrespective of the number of people in an image. The benefit of using this method [25] is that it works reliably regardless of people’s orientations to the camera. However, it should be noted that any other method for finding characteristic points can be used as long as it provides reliable data on point position in social HRI scenarios.

At the next step, the distance to the person *d* is calculated by measuring a Euclidean Distance $D_{et}$ between the characteristic points in the image plane. When the scene is observed through the robot’s camera in social HRI, person(s) in the scene can be oriented in different directions. Therefore, the characteristic points must be observable from all (or the largest number possible) different orientations. In this work, we consider a torso point and one of the ears (the ear that is detected first in the image) as characteristic points. These points can be identified reliably regardless of a person’s orientation using the algorithm reported upon in [25].

The Euclidean distance $D_{et}$ between the characteristic points in the image plane is measured in pixels. We assume that this distance is a predictor of the real distance *d* from the camera to the person, although the exact coefficients of the relationship between the two measures depend on the camera’s sensor and lens. If this assumption can be statistically confirmed, then the proof will also provide the necessary coefficients (namely *a*, *b*, and *k* in Equation (1)) for a particular camera (a combination of a sensor and a lens).

Finally, since the method is based on measurements on pixels in the image space, the method accounts for distance noise. The validation and results are presented further and for statistical analysis, *p* < 0.05 was considered significant.

## 4. Validation

To validate the method, we use a curve-fitting approach based on the assumed relationships stated in Equation (1) in combination with the analysis of monotonicity using Spearman correlations. Therefore, the experimental validation requires the simultaneous capturing of images along with measurements of the ground-truth distances. For this process, Asus Xtion Pro sensor (https://www.asus.com/3D-Sensor/Xtion_PRO/) is used. The sensor provides color and depth images. The color image is used for the distance estimation using the method proposed in Section 3. The depth image is used to extract the actual distance to people in the scene. To obtain reliable depth measurements, the depth sensor is calibrated using the method reported in [26]. Also, the color image is always aligned to the depth image to allow for pixel-to-pixel coordinate mapping between the two. The main reason behind calibration of the camera is to align the RGB image with respect to depth image to obtain reliable distance measurement from the depth image when the pixel is selected on the RGB image. The calibration helps in accumulating reliable ground-truth information and is no way concerned with the actual distance estimation process. The distortion of the camera is also corrected before the start of the experiment. The Xtion Pro sensor is specified by the manufacturer provides accurate distances up to 3.5 m but still provides depth information up to 6 m. Noise analysis of the depth sensor is conducted to account for possible errors in the measurements.

The validation process, which is further explained in Section 4.1, Section 4.2, Section 4.3, Section 4.4 and Section 4.5, is split into several parts:

1. Depth sensor noise analysis: estimates the amount of noise given by the individual pixel depth measurement.
2. Natural distance noise analysis: looks at how much noise can be present on the distance measurement based on the depth sensor when the person is behaving naturally in a conversation scenario.
3. Distance data collection using the proposed method with simultaneous measurements using the depth sensor.
4. Regression of depth-based and image-based distance based on Equation (1).
5. Distance alignment.

Distance data collection is performed in a natural scenario: the sensor is placed in a crowded open space in which the subjects are not constrained to stay in some particular positions. However, an additional dataset in which subjects are placed at predefined distances, is required to perform noise analysis.

### 4.1. Depth Sensor Noise Analysis

Sensor noise analysis is required to assess the amount of noise that is caused by the depth measurement sensor itself. This is done by looking at distance measurement using a single pixel measurement in the depth channel, versus using a mean value of a 5 × 5 pixel patch around the target point. This allows determination of the effect of the noise caused by the sensor, if there is any.

### 4.2. Natural Distance Noise and Error Analysis

Distance measurement noise occurs naturally in conversation scenarios. While we assume that the distance to a person within a scene is the distance to where a person is standing, a person involved in interaction can move constantly. The movements cause noise while measuring immediate distance.

In this study, the fact that distance is measured using a depth sensor is introducing additional noise. The depth sensor measures a range from the camera to a point on a person. This point can be located at the chest, back or a shoulder and be significantly different from the true distance *d*. Therefore, the characteristics of this natural noise needs to be analyzed and accounted for in the results.

The analysis of natural distance noise and errors are performed on a laboratory-based data collection in which subjects were asked to interact with an experimenter when moving around freely (rotating) at three predefined distances from the sensor. The three key distances selected were: 1.5 m, 3.0 m, and 4.5 m. In addition, a D’Agostino-Pearson normality test [27] of the noise dataset at the three predefined distances was conducted. This allowed for further parametric testing.

### 4.3. Distance Data Collection

For the main distance data collection, the sensor is placed in an open crowded space in which the subjects are not constrained by conversation scenarios or specific configurations of the space. The distance data collection setup should allow for natural interaction and not interfere with what the subjects are doing. The data is collected continuously for the duration of several hours.

### 4.4. Regression of Depth-Based and Image-Based Distance Based on Equation (1)

We expect that the results of the data analysis demonstrate a correlation between the Euclidean distance $D_{et}$ between an ear and a torso point and the distance *d* between the camera and a person. We expect that the dependency between the two measures is described by Equation (1). Therefore, a linear regression approach is used to predict the relationship between them. Additionally, a non-parametric analysis of monotonicity is conducted using Spearman’s correlations.

### 4.5. Distance Alignment

In the final step, distance alignment is performed to relate the measured Euclidean distance $D_{et}$ to the real distance *d* between the camera and a person. This process is needed to minimize the effect of depth sensor noise described in Section 4.2. Therefore, the distance alignment is performed using the average values of measured distances at the three key distances from the data collection made in laboratory settings.

## 5. Results

Three datasets have been collected for the purpose of developing the method proposed in this paper: one laboratory-based dataset containing distance measures at three key distances at which subjects stood still or moved around freely (rotating) on the spot, one laboratory-based dataset with distance measures from three predefined distances collected during a conversation with an experimenter, and one dataset with distance measurements collected in a crowded open space.

The main objective of this study is to validate that the ear-torso distance in the image plane is a predictor of a real distance from the camera to a subject. The dataset in a crowded open space was collected for this purpose. Supportive datasets with subjects placed in the predefined spots help to identify the amount of noise that comes from the depth sensor itself, and noise from the moving (interacting) subjects.

### 5.1. Sensor Own Noise Analysis (Pixel vs. Patch)

**Figure 4:**
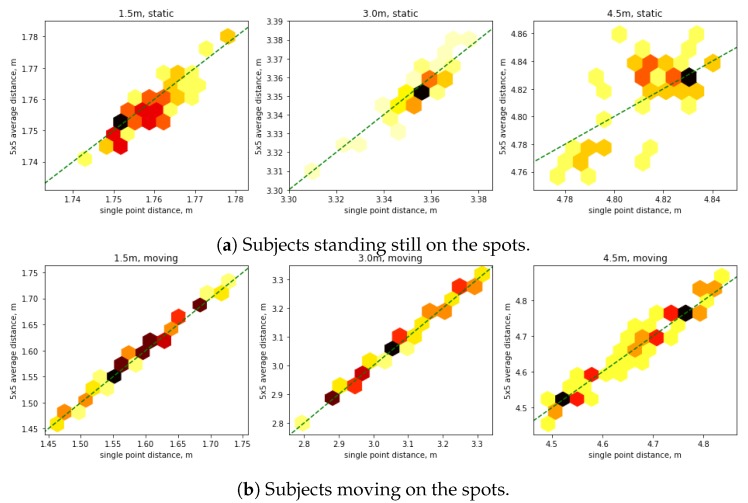
Scatter graphs of the depth sensor noise. Each scatter graph shows the distance measurements using single pixel depth measurement (horizontal axis), meters versus distance measurement using 5 × 5 pixels average depth measurement (vertical axis), meters. Dashed lines represent ideal linear dependency between measurements. The darker colors represent higher density of measurements.

To evaluate the level of noise caused by the depth sensor, a dataset containing data collected with five subjects at predefined distances in a laboratory setting was developed. The data was collected at three key distances: 1.5 m, 3.0 m, and 4.5 m. The collected data considers subjects standing still on the spots. The dataset consists of 172 samples: 61, 59, and 52 in three distance points, respectively. The results for the three key distances are shown in the graphs in Figure 4a.

The graphs show elevated noise at 4.5 m distance. However, the analysis of noise caused by the depth sensor itself is within 2.5% even for the worst noise measured.

### 5.2. Natural Distance Noise and Error Results

The dataset for noise analysis with moving subjects consists of 173 samples. Subjects were instructed to stay on the stops; however, they were allowed to move while interacting with the experimenter. For the moving around freely while rotating, 64, 54, and 55 samples have been collected for the three distances, respectively. The results for the three key distances are shown in the graphs in Figure 4b.

The analysis of datasets in predefined spots allows us to conclude that most of noise in the measurements comes from moving subjects. Even when the subjects were instructed to stand still, they make small natural movements, which is captured by the sensor. This can be observed in Figure 4 that single point measurements strongly correlate with 5 × 5 patch measurements and are located along the ideal correlation lines. Exception is the spot at 4.5 m, which has pronounced own sensor noise, due to sensor physical properties.

#### 5.2.1. Distance Error

For the natural noise analysis, the data has been collected in the laboratory environment. Subject were asked to interact with an experimenter while the distance between them and the camera has been measured using depth sensor and the proposed method. During the interaction, the subjects were asked to move around freely (rotating) on the spot. In total, nine subjects participated in the data collection. In total, 897, 880, and 881 samples have been collected from the three key distances, respectively. The results are shown in Table 1.

The results demonstrate that there is a significant difference between the true distance *d* and the depth sensor reading caused by the interaction noise. This distance must be taken into account by adjusting the final measurements for the value of the difference. In the current study, the distance error between the true and the measured distance is on average +3.5% (the measured distance is bigger than the true distance).

#### 5.2.2. Normality Testing

**Figure 5:**
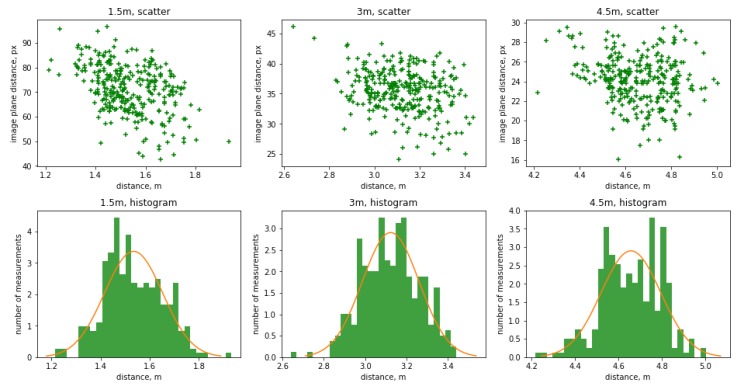
Scatter graphs and histograms for normality testing of the sensor measurements at the three key distances. The graphs in the top row show the distance in meters (horizontal axis) versus pixels (vertical axis). The graphs in the bottom row shows the number of bins (horizontal axis) versus frequency (vertical axis). All measurements performed with interacting subjects. Red line represents ideal normal distribution given the μ and σ of the populations.

The results of the D’Agostino-Pearson normality testing shows the *p*-values p=0.32, p=0.55, and p=0.19 at the three key distances 1.5 m, 3.0 m, and 4.5 m respectively. Therefore, the null hypothesis of normal distribution of the data cannot be rejected. Scatter charts and histograms are presented in Figure 5.

### 5.3. Distance Data Collection

For data collection regarding the assumption “The Euclidean distance between an ear and a torso point in the image space is related to the distance from a camera to the person”, the Xtion Pro sensor was placed in a narrow corridor at the campus of Örebro University. The corridor is usually crowded during the weekdays and the data collection took place between 10:00 and 14:30 on a weekday. To avoid collecting too many samples from the same person(s), the sensor was set to sample one frame every two seconds. The process included: obtain a frame, wait for two seconds then acquire next frame. In the wait time, the obtained frame is processed, and three information’s are written into a text document i.e., a time stamp, the calculated Euclidean distance between characteristic points and the distance obtained using the depth sensor of each subject in the frame.

**Figure 6:**
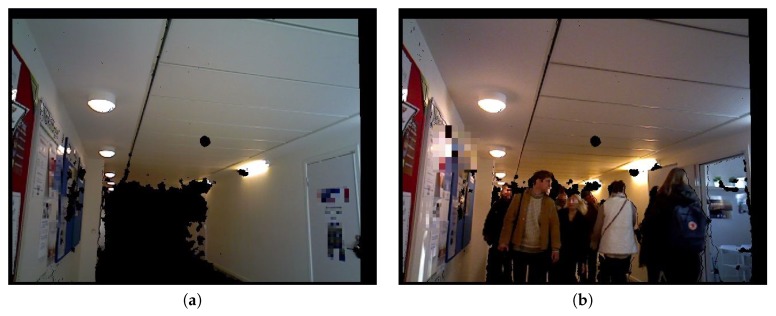
Sample images from distance data collection using the camera (Xtion Pro sensor). The faces of the people are blurred. (**a**) The corridor (**b**) People passing by the camera.

Given that the range was limited to 6 m, less than 5.99 m was considered. One person could be captured on average maximum three times given the average walking speed of 1.5 m/s. Overall, 2120 samples were collected. Each sample includes a time stamp, an estimate of the Euclidean distance $D_{et}$ between each subject’s ear and torso point, and the distance measured using the depth sensor. The depth sensor distance measure was determined by using the depth value of the point in the depth image which corresponds to the previously identified torso point in the color image. As reported upon in Section 3, the torso point was identified by applying the method in [25]. This was possible because the sensor was calibrated to provide pixel-to-pixel matching between the two images. Sample images from the distance data collection are shown in Figure 6.

**Figure 7:**
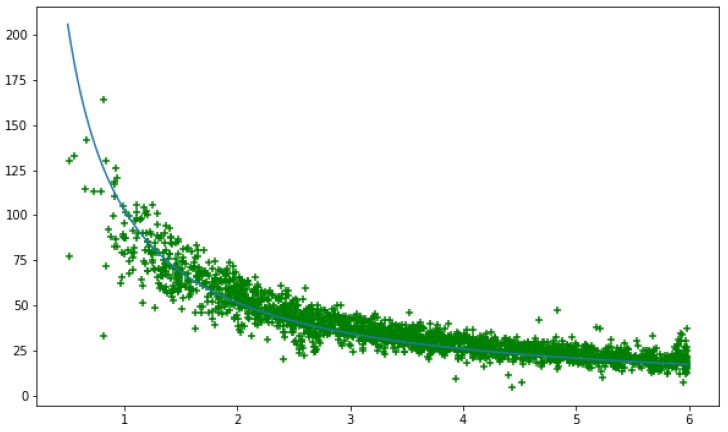
Scatter chart of distance measured using depth data (horizontal axis), meters vs ear-torso Euclidean distance (vertical axis). The blue line is the linear regression based on Equation (1). The distance was recorded for less than 5.99 m. One frame every two seconds was collected to avoid too many samples from the same person. Overall, 2120 samples were collected, and people were walking freely in the corridor.

Figure 7 shows the scatter graphs of the experimental data. The Spearman’s test shows that there is a strong negative correlation between the distances measured using the two methods. The results are statistically significant with the Spearman’s coefficient $r_{S}=-0.9264$ and ρ=0.0.

The linear regression was conducted using Equation (1). The fitted curve is shown as a blue solid line in Figure 7. The best fit was achieved with a=0,b=0. The results show that k=102.94 with the standard deviation σ=0.43. The results are statistically significant with ρ<2.2e−16.

The results support our assumption and show that the Euclidean distance $D_{et}$ between an ear and a torso point of a person in the image space is related to the true distance *d* from a camera to the person.

### 5.4. Distance Alignment

**Figure 8:**
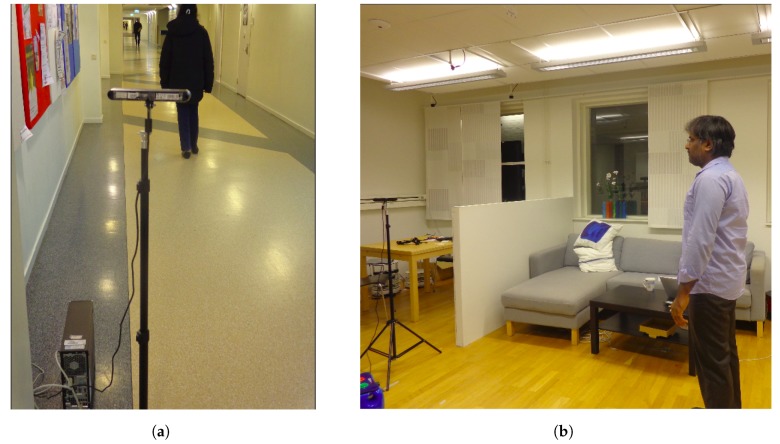
Sample images from data collection using the camera (Xtion Pro sensor). (**a**) The camera setup for data collection in corridor setting to obtain real distance and Euclidean distance between ear and torso points. (**b**) The camera setup for data collection in laboratory setting to obtain depth information with predefined true distance for sensor noise analysis, Table 1.

Because the method for estimating distances is based on measurements on pixels in the image space, the *k* coefficient from Equation (1) will always depend on the exact combination of image sensor and lens. Therefore, a distance alignment needs to be done using a laboratory-based data collection, see Figure 8b. In our study, the depth measured distance to the person can be obtained by dividing the k=102.9426 by the Euclidean distance between an ear and a torso point. Furthermore, the depth measured distance must be aligned to the true distance using the data obtained in Section 5.2.1.

## 6. Discussion and Conclusions

With a growing number of technologies, people are expecting robots to be collaborators, assistants, or social partners that can interact with people in a natural way [28]. To meet this expectation, the first step would be to make robots perceive what is in the scene and “understand” the relationships within the scene. In particular, robots should be aware of the social relationships among humans.

Determining the geometric arrangements, F-formations [2,3], could be the first step in this process and when disassembling the theory of F-formations, estimating distance is a primary requirement. While social and telepresence robots have a camera which could capture RGB images to perceive the scene, few are equipped with distance estimation sensors. There are also a few social robots which are equipped with a depth camera additional to a RGB camera and other sensors. This setup results in a non-trivial process in which the robots need to operate different sensors simultaneously for acquiring RGB images of the scene and getting depth information of people involve in the social interaction.

Vision is the primary sense used by humans to perceive the scene and to understand social interaction. The same ability could also be forged onto robots, i.e., endowing robots with a social skill which allows them to perceive and understand social interaction based on information captured via RGB images which could also be more reliable with multi-sensor fusion.

In this paper, we have proposed and experimentally validated a novel method for estimating the distance between the camera and people in the scene using a single RGB image. The method is insensitive to people’s pose and does not require the input of prior information about camera parameters or features within the scene. Within this paper, we have shown that the Euclidean distance between an ear and a torso point in the image space is related to the distance from a camera to the person. Furthermore, this paper has demonstrated how the Euclidean distance can be converted and provide an estimation of the distance between the person and the camera using Equation (1).

In future works, we aim to endow a robot with a social skill allowing it to perceive and understand social interactions using single RGB images. There are many methods for estimating the head pose, orientation of people from a single image. Combining this information with the distance from the camera to the people and between people would result in the ability of estimating the existence of groups within the scene (image). Endowing a robot with such a social skill could be further extended and allow robots to join groups of people to interact socially with them. 

## Figures and Tables

**Table 1:** Characteristics of the distance error measurements using a depth sensor relative to the true distance at which the subjects had been placed.

	1.5 m	3.0 m	4.5 m
Average distance (μ), m	1.53	3.12	4.67
Standard deviation (σ), m	0.12	0.14	0.13

## References

[1] Hall E. (1966). The Hidden Dimension: Man’s Use of Space in Public and in Private.

[2] Kendon A. (1990). 7 Spatial organization in social encounters: The F-formation system. Conducting Interaction: Patterns of Behavior in Focused Encounters.

[3] Kendon A., Esposito A., Campbell N., Vogel C., Hussain A., Nijholt A. (2010). Spacing and orientation in co-present interaction. Development of Multimodal Interfaces: Active Listening and Synchrony, Second COST 2102 International Training School.

[4] Pathi S.K., Kiselev A., Loutfi A. Estimating f-formations for mobile robotic telepresence. Proceedings of the 12th ACM/IEEE International Conference on Human-Robot Interaction (HRI 2017).

[5] Kristoffersson A., Coradeschi S., Loutfi A. (2013). A Review of Mobile Robotic Telepresence. Adv. Hum.-Comput. Interact..

[6] Shoani M.T.A., Amin S.H., Sanhoury I.M. Determining subject distance based on face size. Proceedings of the 10th Asian Control Conference: Emerging Control Techniques for a Sustainable World, ASCC 2015.

[7] Wang T.H., Hsu C.C., Chen C.C., Huang C.W., Lu Y.C. Three-dimensional measurement of a remote object with a single CCD camera. Proceedings of the ICARA 2009—4th International Conference on Autonomous Robots and Agents.

[8] Gao X., Zheng M., Meng M.Q.H. Humanoid robot locomotion control by posture recognition for human-robot interaction. Proceedings of the 2015 IEEE International Conference on Robotics and Biomimetics (ROBIO).

[9] Di Nuovo A., Conti D., Trubia G., Buono S., Di Nuovo S. (2018). Deep learning systems for estimating visual attention in robot-assisted therapy of children with autism and intellectual disability. Robotics.

[10] Benet G., Blanes F., Simó J.E., Pérez P. (2002). Using infrared sensors for distance measurement in mobile robots. Robot. Auton. Syst..

[11] Saad M.M., Bleakley C.J., Dobson S. (2011). Robust high-accuracy ultrasonic range measurement system. IEEE Trans. Instrum. Meas..

[12] Xing G., Tian S., Sun H., Liu W., Liu H. People-following system design for mobile robots using kinect sensor. Proceedings of the 2013 25th Chinese Control and Decision Conference (CCDC).

[13] Alletto S., Serra G., Calderara S., Cucchiara R. (2015). Understanding social relationships in egocentric vision. Pattern Recognit..

[14] Bianco S., Buzzelli M., Schettini R. (2019). A unifying representation for pixel-precise distance estimation. Multimed. Tools Appl..

[15] Lau B., Arras K.O., Burgard W. (2010). Multi-model hypothesis group tracking and group size estimation. Int. J. Soc. Robot..

[16] Mccoll D., Zhang Z., Nejat G. (2011). Human body pose interpretation and classification for social human-robot interaction. Int. J. Soc. Robot..

[17] Yun S.S., Kim M., Choi M.T. (2013). Easy Interface and Control of Tele-education Robots. Int. J. Soc. Robot..

[18] Komatsubara T., Shiomi M., Kaczmarek T., Kanda T., Ishiguro H. (2019). Estimating Children’s Social Status Through Their Interaction Activities in Classrooms with a Social Robot. Int. J. Soc. Robot..

[19] Yan H., Ang M.H., Poo A.N. (2014). A Survey on Perception Methods for Human-Robot Interaction in Social Robots. Int. J. Soc. Robot..

[20] Nedevschi S., Schmidt R., Danescu R., Frentiu D., Marita T., Graf T., Oniga F., Pocol C. High accuracy stereo vision system for far distance obstacle detection. Proceedings of the IEEE Intelligent Vehicles Symposium.

[21] Suh Y.S., Phuong N.H.Q., Kang H.J. (2013). Distance estimation using inertial sensor and vision. Int. J. Control Autom. Syst..

[22] König I., Beau P., David K. A new context: Screen to face distance. Proceedings of the International Symposium on Medical Information and Communication Technology.

[23] Burgos-Artizzu X.P., Ronchi M.R., Perona P., Fleet D., Pajdla T., Schiele B., Tuytelaars T. (2014). Distance estimation of an unknown person from a portrait. Proceedings of the European Conference on Computer Vision, ECCV 2014.

[24] Todd J.T., Mark L.S., Shaw R.E., Pittenger J.B. (1980). The perception of human growth. Sci. Am..

[25] Cao Z., Simon T., Wei S.E., Sheikh Y. Realtime multi-person 2D pose estimation using part affinity fields. Proceedings of the 30th IEEE Conference on Computer Vision and Pattern Recognition, CVPR 2017.

[26] Teichman A., Miller S., Thrun S. Unsupervised Intrinsic Calibration of Depth Sensors via SLAM. Proceedings of the Robotics: Science and Systems IX.

[27] D’Agostino R., Pearson E.S. (1973). Tests for departure from normality. Empirical results for the distributions of *b*_2_ and √*b*_1_. Biometrika.

[28] Breazeal C. (2004). Social interactions in HRI: The robot view. IEEE Trans. Syst. Man Cybern. Part C Appl. Rev..

